# FOXQ1 regulates senescence-associated inflammation via activation of SIRT1 expression

**DOI:** 10.1038/cddis.2017.340

**Published:** 2017-07-20

**Authors:** Pan Wang, Cuicui Lv, Tao Zhang, Junling Liu, Jin Yang, Fangxia Guan, Tianpei Hong

**Affiliations:** 1 Department of Endocrinology and Metabolism, Peking University Third Hospital, Beijing, China; 2 Department of Clinical Laboratory, Key Clinical Laboratory of Henan Province, The First Affiliated Hospital of Zhengzhou University, Zhengzhou, China; 3 Department of Clinical Laboratory, The Second Affiliated Hospital of School of Medicine, Zhejiang University, Hangzhou, China; 4 School of Life Sciences, Zhengzhou University, Zhengzhou, China

## Abstract

Cellular senescence is an initial barrier to tumor development that prevents the proliferation of premalignant cells. However, some of the features of senescent cells seem to promote tumor progression via senescence-associated secretory phenotype (SASP). Here, we demonstrated that the protein level of forkhead box Q1 (FOXQ1), which highly overexpresses in several kinds of tumors, was significantly downregulated during both replicative and oncogene-induced senescence. Moreover, overexpression of *FOXQ1* delayed senescence, whereas *FOXQ1* silence led to premature senescence in human fibroblasts. Furthermore, we identified that FOXQ1 upregulated SIRT1 expression through transcriptional regulation via directly binding to the *SIRT1* promoter. Finally, we showed that FOXQ1 remarkably inhibited the replicative senescence through depressing the expression of the inflammatory cytokines interleukin-6 (IL-6) and IL-8 via modulation of SIRT1-NF-*κ*B pathway. In addition, FOXQ1 overexpressed in human esophageal cancer cells and ablation of *FOXQ1* restrained the tumourigenic ability of the esophageal cancer cells (EC109 and EC9706) in a mouse xenograft model *in vivo*. Taken together, these findings uncover a previously unidentified role of FOXQ1 regulating SASP and tumor development at same time.

Cell senescence is a cell cycle arrest program characterized by an irreversible growth arrest in response to diverse forms of cellular stress which renders senescent cells insensitive to mitogenic stimuli and undoubtedly contributes to key senescence phenotypes.^1, 2^ Two pathways, p53/p21 and p16^INK4a^/Rb (retinoblastoma), can orchestrate the development of senescence in response to DNA damage, oxidative stress and oncogene activation.^3^ The senescence response is widely recognized as a potent tumor suppressive mechanism.^4^ However, recent evidence strengthens the idea that senescent cells seem to mediate the development of age-related pathologies, including cancer.^5, 6^ There is mounting evidence that senescent cells, which often persist *in vivo*, can promote tumor progression in addition to other age-related pathologies via the senescence-associated secretory phenotype (SASP).^7, 8^

Senescent cells can secret numerous kinds of pro-inflammatory cytokines, chemokines, growth factors and proteases, which are termed as SASP.^9^ SASP contributes to multiple biological effects depending upon the context. Some SASP factors such as interleukin-6 (IL-6) and IL-8 have been described to reinforce the senescence program in an autocrine manner, and to promote senescence induction in a paracrine mode.^9, 10^ Besides, SASP factors can also promote the immune surveillance of senescent cells by stimulating the immune system to clear premalignant senescent cells and facilitate tissue repair.^11, 12^ Nevertheless, SASP may promote inflammation and exert a pro-proliferative activity, thus contributing to cancer initiation and progression.^13^ SASP is regulated mainly at mRNA level and depends on NF-*κ*B and C/EBP*β* activities.^14^ More recently, it has been shown that IL-1*α* and NF-*κ*B comprise a positive feedback loop that stimulates the transcription of SASP factors.^15^ Additionally, miR-146 a/b, Klotho, protein kinase D1 and GATA4 have also been implicated as a regulator of SASP.^16, 17, 18, 19^ Overall, though great progresses have been made in investigating the molecular mechanisms of SASP, there is still a long way to go to illuminate the role of SASP in aging process.

Forkhead box (FOX) family proteins, which characterized by presence of an evolutionarily conserved DNA binding domain, involve in a wide range of crucial cellular processes including stress resistance, metabolism, cell cycle arrest, aging and apoptosis.^20^ Foxq1 was first found in modulating hair follicle development in mammals,^21^ and was also noticed in regulating the major stomach mucin expression and granule content in stomach surface mucous cells in rodents.^22^ Thereafter, evidence indicated that FOXQ1 highly expressed in colorectal cancer, breast cancer, liver cancer, esophageal cancer, non-small cell lung cancer and other cancers.^23, 24, 25, 26, 27, 28, 29^ It has been reported that miR-320 can suppress colorectal cancer by targeting *FOXQ1*.^30^ Moreover, FOXQ1 can promote hepatocellular carcinoma metastasis via activating the transcription of ZEB2 and versican V1.^31^ However, its role in longevity remains to be elucidated.

In the present study, we demonstrated that FOXQ1 expression level was downregulated during both replicative and oncogene-induced senescence. We characterized that in human 2BS diploid fibroblasts overexpression of *FOXQ1* could promote cell growth, while *FOXQ1* silencing lead to premature senescence. We also identified that FOXQ1 could downregulate IL-6 and IL-8 expression through SIRT1-mediated inhibition of inflammatory pathway. Moreover, we showed that FOXQ1 protein level was elevated in human esophageal cancer cells and the tumourigenic ability of FOXQ1 in the cancer cells was confirmed in a mouse xenograft model *in vivo*. Our findings may allow more effective approaches to the cancer treatment by inhibiting the expression of FOXQ1 and inducing cell senescence.

## Results

### Expression of FOXQ1 is decreased during replicative senescence and oncogene-induced senescence

**Figure 1:**
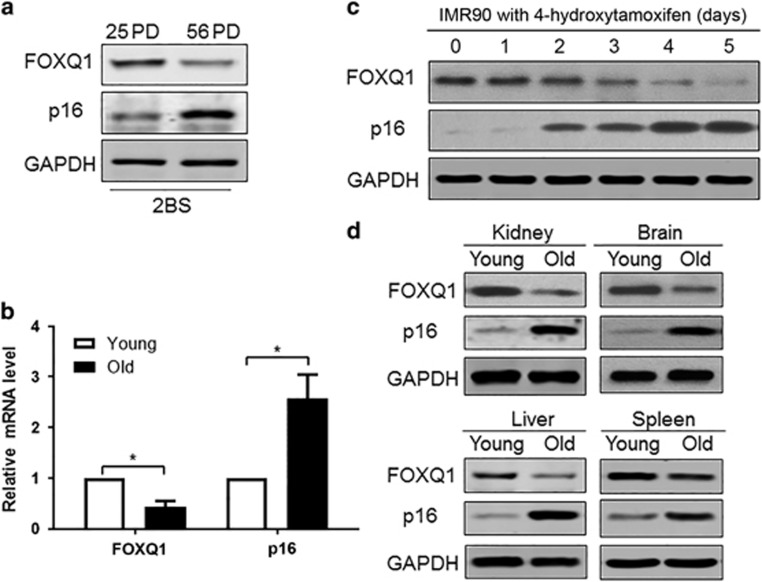
Expression of FOXQ1 decreases with senescence. (**a**) Western blot analysis of FOXQ1 and p16^INK4a^ level in 25 PD and 56 PD 2BS cells. (**b**) qRT-PCR analysis of *FOXQ1* and *p16*^*INK4a*^ in the young and old 2BS cells. The mRNA expression levels of indicated gene were normalized to *β*-actin. Each bar represents the means±S.D. for triplicate experiments. *P<0.05. (**c**) FOXQ1 was time-dependently downregulated in IMR90 cells with tamoxifen-induced Ras expression. Protein level of FOXQ1 and p16^INK4a^ at different times following 4-hydroxytamoxifen treatment was determined by western blot analysis. (**d**) Protein levels of FOXQ1 in indicated tissues of young (3 months) and old (18 months) BALB/c mice were determined by western blot analysis. GAPDH was used as loading control

In view of the positive effect of FOXQ1 on the proliferation and metastasis of cancer cells, we speculated that FOXQ1 might also be involved in cellular senescence. Therefore, we first evaluated FOXQ1 expression in the young and senescent human 2BS fibroblasts and in the Ras oncogene-induced premature IMR90 cells. Western blot results showed that the protein level of FOXQ1 was lower in the senescent 2BS cells than that in the young cells. In contrast, the protein level of p16^INK4a^ was higher in the senescent cells than that in the young cells (Figure 1a). Likewise, quantitative reverse transcription polymerase chain reaction (qRT-PCR) analysis demonstrated downregulation of *FOXQ1* and upregulation of *p16*^*INK4a*^ transcripts in the aged fibroblasts (Figure 1b). To consolidate this observation, we also assessed their protein levels in human IMR90 diploid fibroblasts that were stably integrated with a tamoxifen-regulated form of activated Ras. In line with the above findings in the senescent 2BS cells, we observed a marked reduction of FOXQ1 protein level accompanied by an increased p16^INK4a^ protein level after exposed to 4-hydroxytamoxifen in a time-dependent manner (Figure 1c).

In order to clarify whether the expression profiles of FOXQ1 and p16^INK4a^ with aging could be recapitulated *in vivo*, we examined their protein level in several tissues from young (3 months) and old (18 months) adult BALB/c mice. The results showed that there was a significant decrease of FOXQ1 level and increase of p16^INK4a^ level with age in kidney, brain, spleen and liver (Figure 1d). Collectively, this cell passage- or animal age-dependent reduction of FOXQ1 level suggested that FOXQ1 might be involved in the process of cellular senescence.

### *FOXQ1* overexpression delays cellular senescence, whereas *FOXQ1* silencing leads to premature senescence in human fibroblasts

**Figure 2:**
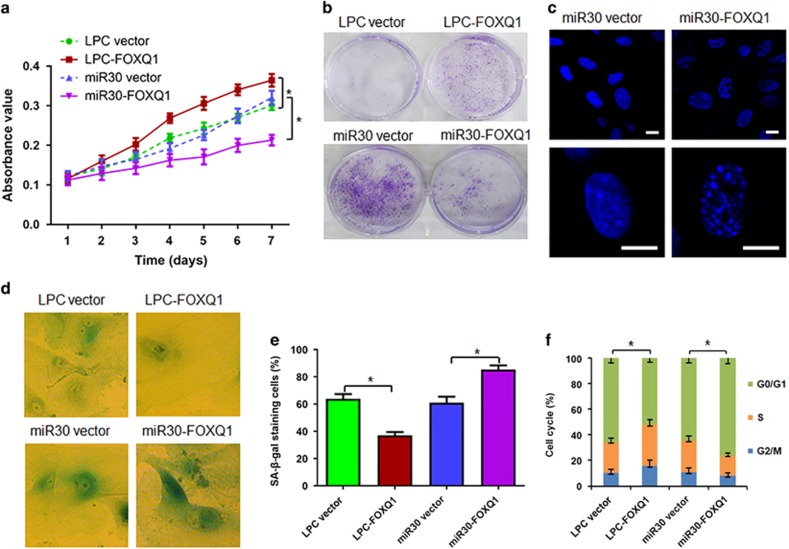
*FOXQ1* overexpression promotes cell proliferation and *FOXQ1* silence causes premature senescence. (**a**) The 2BS cells expressing the indicated genes and shRNAs were cultured and growth curves were determined by the MTT assay. (**b**) The 2BS cells were infected with indicated retroviruses and cultured in the 6-well plates for 6 days, followed by fixation and staining with crystal violet. (**c** and **d**) Representative images of the indicated cells with stained for senescence-associated heterochromatin foci by DAPI (**c**) and SA-*β*-gal activity (**d**) were shown (Scale bar, 10 *μ*m). (**e**) The percentage of cells positive for SA-*β*-gal in each sample was determined. At least 300 cells were counted for each sample. (**f**) Cell cycle was measured. Values are means±S.D. of triplicate points from a representative experiment, which was repeated three times with similar results. *P<0.05

To determine the functional role of FOXQ1 in the cell senescence, *FOXQ1* was overexpressed and silenced, respectively, with a retrovirus expression system in the 2BS cells. Cell proliferation and senescence markers were then monitored at several time points. Growth curve and crystal violet staining assays indicated that the 2BS cells with ectopic *FOXQ1* expression displayed higher proliferation rate (Figure 2a) and more colony formation (Figure 2b) than those in the cells with empty vector. Next, we took a short hairpin RNA (shRNA)-based knockdown approach to examine the requirement of FOXQ1 for senescence progression. In concert with the effect of ectopic *FOXQ1* expression, its removal resulted in lower proliferation rate (Figure 2a) and less colony formation (Figure 2b) than those in the cells transduced with empty vector. Meanwhile, miR30-*FOXQ1* also resulted in emerging the morphological features of senescence, characterized by enlarged and flattened cell size, increased senescence-associated heterochromatin foci (Figure 2c), elevated activity of senescence-associated *β*-galactosidase (SA-*β*-gal), a biomarker for senescent cells (Figures 2d and e), and reduced S and increased G1 compartments (Figure 2f) compared with scramble control vector. Conversely, LPC-*FOXQ1* induced much lower SA-*β*-gal activity (Figures 2d and e) and less cell cycle progression (Figure 2f) than the infection with its corresponding empty control vector. These results demonstrated that FOXQ1 was essential for replicative cell senescence and that *FOXQ1* overexpression promoted human fibroblast proliferation, whereas *FOXQ1* silencing induced the cell senescence.

### FOXQ1 increases the mRNA and protein levels of SIRT1

**Figure 3:**
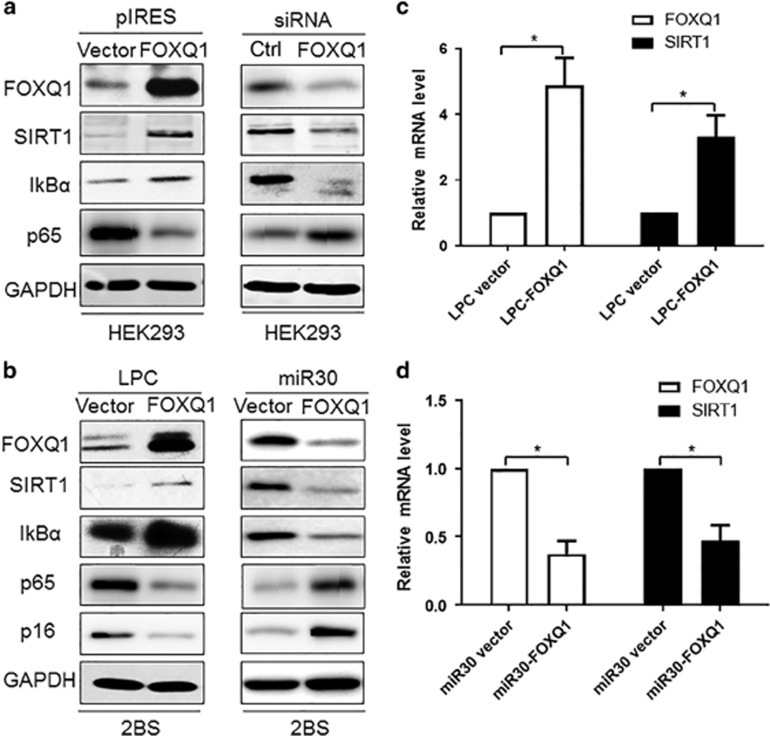
FOXQ1 upregulates SIRT1 at mRNA and protein levels. (**a**) Western blot analysis of FOXQ1, SIRT1, IkB*α* and p65 level was carried out in *FOXQ1* overexpression or knockdown cells compared with control cells. HEK293 cell was used for these experiments. (**b**) The 2BS cells were stably transduced LPC-*FOXQ1*, miR30-*FOXQ1* or control vector with retrovirus. The infected 2BS cells were selected and then the indicated proteins were determined by western blot. (**c** and **d**) The mRNA expression level of *FOXQ1* and *SIRT1* were determined qRT-PCR. Data represent the means±S.D. for triplicate experiments. *P<0.05

As mentioned above, our results showed that *FOXQ1* overexpression promoted cell growth, whereas *FOXQ1* knockdown led to growth inhibition. We therefore examined the molecular mechanism by which FOXQ1 delayed cellular senescence. As SIRT1 is an important determinant of longevity that regulates lifespan in diverse species,^32^ and mounting evidences support the concept that SIRT1 is an essential regulator of inflammation by altering histones and transcription factors such as NF-*κ*B,^33^ we thus investigated whether FOXQ1 could mediate cell senescence via directly regulating SIRT1. Western blot analysis revealed that *FOXQ1* overexpression significantly increased the protein level of SIRT1 in HEK293T cells (Figure 3a). In addition, we silenced the *FOXQ1* gene and examined SIRT1 protein levels by western blot. As expected, we noticed that a markedly decreased level of SIRT1 protein in the *FOXQ1* siRNA-transfected cells compared with the control scrambled siRNA-transfected cells (Figure 3a). Meanwhile, the protein levels of p65RelA and I*κ*B*α* were also analyzed by western blot. We found that the protein level of I*κ*B*α*, an inhibitor of NF-*κ*B, positively correlated with FOXQ1 and SIRT1, while the level of p65RelA displayed an opposite tendency with FOXQ1 and SIRT1 (Figure 3a). To further strengthen this argument, we overexpressed *FOXQ1* by pLPC-Puro retroviral vector and silenced it using miR30 retroviral vector in the 2BS cells. We observed the identical results to those from HKE293 cells (Figure 3b). Besides, increased *FOXQ1* expression resulted in a decreased level of p16^INK4a^ protein, while removal of *FOXQ1* exhibited an opposite effect on p16^INK4a^ protein level (Figure 3b). In parallel with the findings from western blot analysis, qRT-PCR analysis also showed a positive regulation of *SIRT1* mRNA expression by FOXQ1 (Figures 3c and d), indicating that FOXQ1 could activate transcription of *SIRT1*.

### FOXQ1 promotes proliferation through suppressing IL-6 and IL-8 expression via transcriptional upregulation of SIRT1

**Figure 4:**
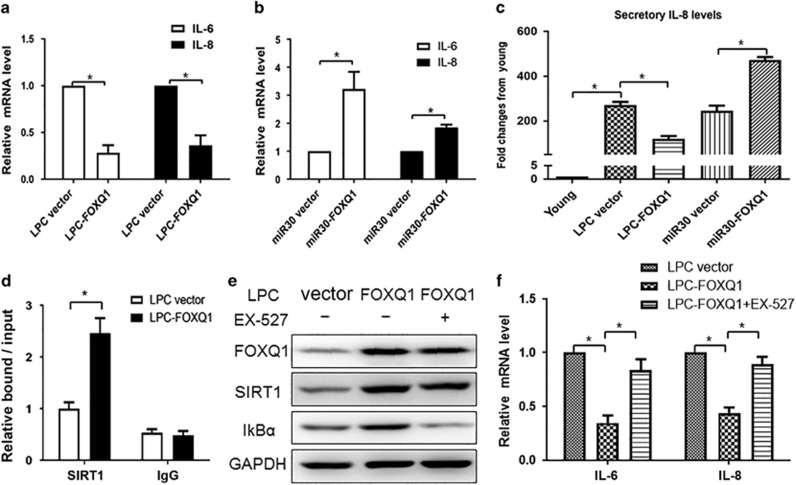
Regulation of IL-6 and IL-8 expression by FOXQ1 is dependent on SIRT1 activity. (**a-c**) Ectopic expression of *FOXQ1* inhibited, and *FOXQ1* silencing promoted IL-6 and IL-8 expression. The 2BS cells stably transduced with LPC-*FOXQ1* (**a**) or miR30-*FOXQ1* (**b**) were selected and cultured for three days. Total RNA was extracted from the 2BS cells expressing the indicated genes and the relative mRNA level of IL-6 and IL-8 were determined by quantitative PCR. Supernatants collected from the above cells were used to assess secretory levels of IL-8 by ELISA (**c**). The IL-8 level was standardized with that of the young cells. (**d**) FOXQ1 could bind to the promoter of *SIRT1*. The 2BS cells were stably transduced with the LPC-*FOXQ1* and control vectors. After selection, the cell lysates were analyzed by ChIP assay using an antibody against FOXQ1. FOXQ1 binding to the *SIRT1* promoters is represented as level relative to mouse IgG binding. (**e**) SIRT1 inhibitor EX-527 abolishes the FOXQ1-mediated I*κ*B*α* upregulation. The 2BS cells expressing LPC-*FOXQ1* were treated with EX-527 (10 *μ*M) for 24 h, and then the cells were harvested and analyzed for the level of indicated proteins. (**f**) EX-527 eliminated the FOXQ1-induced inhibition of IL-6 and IL-8 transcriptional activity. Total RNA was extracted from the cells as indicated in (**e**) and the relative mRNA levels of IL-6 and IL-8 were determined by quantitative PCR. Error bars represent means±S.D. *n*=3. *P<0.05

It is well known that SIRT1 is a key regulator of inflammation in mammalian cell via inhibition of NF-*κ*B activity. Based on our above observation that FOXQ1 could increase the protein levels of both SIRT1 and I*κ*B*α*, we prudently inferred that FOXQ1 might regulate cell senescence via modulating senescence-associated inflammation. To further substantiate the inference, we performed experiments with gain- and loss-of-function of *FOXQ1* in the 2BS cells. As IL-6 and IL-8 are both required for the inflammatory network and senescence entry and maintenance, we tested whether FOXQ1 could regulate IL-6 and IL-8 expression via modulating SIRT1 activity. Here we used qRT-PCR to determine the mRNA levels of *IL-6* and *IL-8*. LPC-*FOXQ1* markedly decreased the mRNA abundance of *IL-6* and *IL-8* compared with control transfection (Figure 4a). In contrast, *FOXQ1* deletion significantly elevated *IL-6* and *IL-8* mRNA levels (Figure 4b). Moreover, the secreted levels of IL-8 in supernatant detected by enzyme linked immunosorbent assay (ELISA) showed similar alteration with its mRNA level (Figure 4c). All together, these results indicated that FOXQ1 was an important mediator in SASP by regulating IL-6 and IL-8 induction.

We next sought to explore the molecular mechanism underlying the FOXQ1-mediated transcriptional activation of SIRT1. To test the possibility that FOXQ1 directly bound to the *SIRT1* promoter region, we performed a chromatin immunoprecipitation (ChIP) assay followed by quantitative PCR (ChIP-qPCR). The ChIP-qPCR results indicated a significant enrichment of FOXQ1 in the promoter of *SIRT1* in the *FOXQ1* overexpressed cells compared with the control cells (Figure 4d). To investigate the functional importance of the FOXQ1-SIRT1 axis in SASP, we used a pharmacological method to clarify whether the regulation of IL-6 and IL-8 expression by FOXQ1 was dependent on SIRT1 activity. We performed an epistasis analysis by overexpression of *FOXQ1* with or without EX-527, a specific SIRT1 inhibitor. Western blot results revealed that the FOXQ1-induced upregulation of I*κ*B*α* level was abolished by addition of EX-527 treatment (Figure 4e). In addition, real-time PCR results demonstrated that *FOXQ1* overexpression led to the decrease of *IL-6* and *IL-8* expression levels, which was eliminated by inhibition of SIRT1 activity with EX-527 (Figure 4f). These results suggested that the FOXQ1-mediated regulation of SASP factors was dependent on SIRT1-NF-*κ*B pathway.

### FOXQ1 highly expressed in esophageal cancer cells contributes to cell proliferation *in vitro*

**Figure 5:**
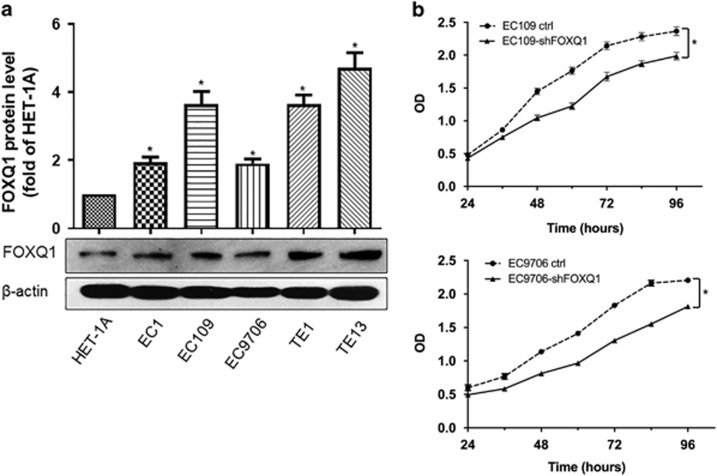
FOXQ1 overexpressed in esophageal cancer cells contributes to tumor cell proliferation *in vitro*. (**a**) Western blot analysis of FOXQ1 protein levels in human esophageal cancer cell lines and the human esophageal epithelial cell line HET-1A. The relative level of FOXQ1 protein is normalized to *β*-actin (loading control). (**b**) EC109 and EC9706 cells stably expressing *shFOXQ1* or control vector were seeded at the same number in each well. The cell growth was monitored by MTT assay every 12 h. Values are means±S.D. of triplicate points from a representative experiment, which was repeated three times with similar results. *P<0.05

It is well known that cellular senescence is a potent tumor suppressive mechanism characterized by an irreversible growth arrest. However, several lines of evidence show that senescent cells can also promote tumor progression via SASP.^7, 13^ In the present study, we showed that FOXQ1 could promote cell proliferation and delay cell senescence by inhibition of IL-6 and IL-8 expression. Therefore, we curiously wanted to know the expression profile of FOXQ1 in cancer cells and its role in the cancer development. To this end, we first measured FOXQ1 protein level in 5 human esophageal cancer cell lines (EC1, EC109, EC9706, TE1 and TE13) and the normal human esophageal epithelial cell line HET-1A. The relative level of FOXQ1 protein was higher in all the esophageal cancer cell lines than that of the HET-1A cells (Figure 5a). Next, we silenced *FOXQ1* by using lentivirus in EC109 and EC9706 cells. The MTT assay demonstrated that silence of *FOXQ1* caused cell growth retardation compared with its corresponding empty vector control (Figure 5b). These findings indicated that FOXQ1 might contribute to the progression of esophageal cancers, which is in agreement with the results of previous studies.^28^

### FOXQ1 affects tumor growth in a mouse xenograft implantation model

**Figure 6:**
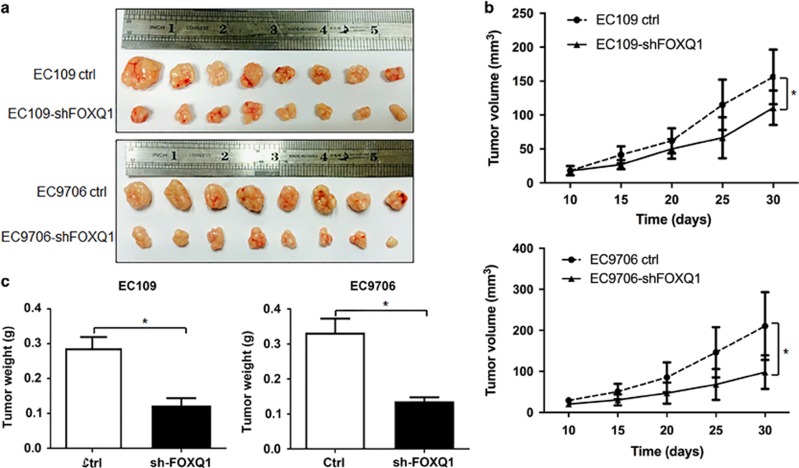
FOXQ1 promotes tumourigenicity *in vivo*. (**a**) *FOXQ1* silence inhibits the xenograft tumor growth of EC109 and EC9706 cells. The cells (1 × 10^6^ cells) stably expressing *FOXQ1* silencing or control lentiviral vector were injected subcutaneously into the left or right flank of NOD/SCID mice, respectively. At 30 days after injection, tumors were excised and photographed. (**b**) Tumor growth curves in mouse xenograft model. Tumor diameters were measured at the indicated time points, and tumor volumes were calculated. (**c**) Tumor weights from the same experiment as above on day 30. Values are means±S.D. *n*=8. *P<0.05

Given that our *in vitro* studies suggested a functional role for FOXQ1 in the esophageal cancer cell proliferation, we next investigated the contribution of FOXQ1 to the esophageal cancer cell growth *in vivo*. To this end, stable EC109 and EC9706 cells transduced with either *FOXQ1* silencing or control lentiviral vector were subcutaneously injected into the flank of NOD/SCID mice, and tumor growth was monitored over a period of 30 days. The mean tumor volume and weight of the *FOXQ1* silencing vector-expressing EC109 and EC9706 xenografts grew at a slower rate than those derived from the xenografts expressing control vector (Figures 6a–c). Taken together, these results suggested that FOXQ1 might promote cell proliferation and tumourigenicity of the esophageal cancer cells *in vivo*.

## Discussion

It has been reported that cellular senescence functions as a general protective mechanism against proliferative stress responses and cancer development *in vivo* early in life, however, late in life senescent cells accumulate in different tissues in aging mammals and seem to mediate the development of age-related pathologies, including cancer.^5^ FOXQ1 has been found to regulate a number of processes including cell proliferation, differentiation and development, especially tumor proliferation.^21, 22, 23, 24, 25, 26, 27, 28, 29^ In this study, we showed that the protein level of FOXQ1 was decreased during cell senescence both *in vitro* and *in vivo*. Moreover, *FOXQ1* overexpression delayed cellular senescence, whereas *FOXQ1* silencing led to premature senescence in human fibroblasts. These results suggest that FOXQ1 is involved in the regulation of cellular senescence. To our knowledge, this is the first report that FOXQ1 can regulate cell senescence except tumor development and metastasis. Most importantly, we deciphered that FOXQ1 modulated cell senescence via positively regulating the NAD-dependent deacetylase SIRT1 at transcriptional level. Given the definite role of SIRT1 in cell senescence, our data identified the undisputed importance of FOXQ1-SIRT1 signaling in cell senescence.

Chronic inflammation has been reported to be the reason of many age-related diseases, including atherosclerosis, heart failure, osteoporosis, neurodegeneration and cancer.^6^ SASP is characterized by being an indispensable constituent of chronic inflammation and is proposed to have important roles in aging and many senile diseases.^13^ However, the causes and consequences of SASP are not very clear. By gain- and loss-of-function studies, we demonstrated that *FOXQ1* overexpression could suppress the expression of some SASP factors such as IL-6 and IL-8, which might account for the effect that FOXQ1 was able to delay cell senescence in the human 2BS fibroblasts. Conversely, *FOXQ1* silencing could stimulate the production of these SASP factors and might thus contribute to the premature senescence. Therefore, we deciphered a previously unidentified role and mechanism of SASP in cell senescence for the first time.

In the present study, we also noticed that FOXQ1 highly expressed in all the 5 human esophageal cancer cell lines. Compared with the corresponding tumor cells transduced with empty control vector, the growth of human esophageal cancer cells was retarded in the *FOXQ1* silencing vector-expressing EC109 and EC9706 cells both *in vitro* and *in vivo*. These findings are in agreement with the previous reports that FOXQ1 is involved in the development and progression of malignant tumors including esophageal cancer.^23, 24, 25, 26, 27, 28, 29^ Therefore, FOXQ1 might potentially act as an important regulator in the maintenance of the balance between cell senescence and tumor development. The different expression level of FOXQ1 might lead to different cell fate as black and white. Furthermore, it has been reported that Foxq1 is restrictedly expressed in rapidly proliferated stomach cells,^22^ which consolidates its role in promoting cell proliferation once more. On the other hand, the degradation of FOXQ1 protein during cell senescence is considerable and the invalidation of this process might be the reason why high level of FOXQ1 protein is found in several kinds of tumor tissues and can thus promote cancer cell proliferation and metastasis.^23, 24, 25, 26, 27, 28, 29^ The mechanism through which FOXQ1 is degraded in cell senescence remain to be identified in the future study. Given that *Foxq1* mutant mice are available,^22^ it can be helpful to use this animal model to investigate tissue and organ senescence in our future work.

SASP is regulated mainly by NF-*κ*B and C/EBP*β*, and SIRT1 is a key regulator of inflammation in mammalian cells mainly through deacetylation of p65, thus inhibiting NF-*κ*B activity.^34^ Here, we found that the level of I*κ*B*α*, an inhibitor of NF-*κ*B, was positively correlated with SIRT1, which implied that the inhibitory effect of SIRT1 on NF-*κ*B activity was mainly dependent on I*κ*B*α* activity in this study. In fact, our study also showed that the *FOXQ1* overexpression-induced upregulation of I*κ*B*α* protein level and downregulation of IL-6 and IL-8 expression were abolished by the specific SIRT1 inhibitor EX-527. These results indicate that SIRT1 may strengthen the inhibitive effect on NF-*κ*B-mediated inflammatory pathway through promoting I*κ*B*α* expression in addition to deacetylation of p65.

In summary, this study demonstrated a previously unknown role of FOXQ1 in cell senescence. Overexpression of *FOXQ1* promoted fibroblast proliferation and upregulated the level of SIRT1 expression. Besides, decreased level of FOXQ1 expression during cell senescence induced IL-6 and IL-8 expression, and subsequently reinforced cell senescence via transcriptional inhibition of SIRT1. In this study, we also identified that FOXQ1 highly expressed in human esophageal cancer cells and that *FOXQ1* silence inhibited the tumor cell growth both *in vitro* and *in vivo*. Therefore, our study uncovers a role and mechanism of FOXQ1 in the regulation of cell senescence, offering new insights on the balance between cell senescence and tumor development.

## Materials and methods

### Plasmids, antibodies, reagents and animals

Full-length *forkhead box Q1* (*FOXQ1*) was cloned from the cDNA of normal human fibroblast using a PCR-based approach and was subcloned into pLPC-pure vector and pIRES vector respectively. The shRNA plasmid was designed and cloned as described.^35^ The primary antibodies used for western blot analysis were as follows: anti-FOXQ1, anti-SIRT1, anti-p16 and anti-*β-*actin were from Santa Cruz Biotechnology (Santa Cruz, CA, USA). Anti-I*κ*B*α* and anti-p65 was purchased from Cell Signaling Technology (Beverly, MA, USA). Anti-GAPDH was from Proteintech Group (Wuhan, China). 4-Hydroxytamoxifen (T5648, Sigma, St. Louis, MO, USA) was dissolved in methanol; and EX-527 (Selleck Chemicals, Shanghai, China) was dissolved in DMSO (D2650, Sigma). BALB/c mice, which were obtained from Beijing Vital River Laboratory Animal Technology Co., Ltd. (Beijing, China), were maintained in a certified animal facility in accordance with the guidelines set forth by the Animal Ethics Committee of the First Affiliated Hospital of Zhengzhou University.

### Cell lines, cell culture and viral infections

Human diploid 2BS fibroblasts (National Institute of Biological Products, Beijing, China) as well as IMR90 human diploid fibroblasts transduced with an ER:Ras (kindly provided by Dr. Masashi Narita, Cancer Research U.K., Cambridge Research Institute) were cultured in Dulbecco’s modified Eagle’s medium supplemented with 10% FBS (HyClone, Logan, UT, USA) and antibiotics. The 2BS cells are considered to be young at 30 PD or less and replicative senescent 2BS cells are defined as >55 PD. To induce senescence, the IMR90 cells expressing ER:H-RasV12 were exposed to 100 nM 4-hydroxytamoxifen. Retroviruses were packed using Phoenix cells and the infections were performed as previously described.^36^ Besides, HET-1A, EC1, EC109, EC9706, TE1 and TE13 cells were maintained in RPMI-1640 medium supplemented with 10% FBS.

### RNA interference

Chemically synthesized double-stranded siRNA was used against the transcript of *FOXQ1*. Cells were transfected with siRNA oligonucleotides for 72 h using Lipofectamine RNAiMAX (Thermo Fisher Scientific, Waltham, MA, USA).

### Quantitative real-time RT-PCR

Total RNA was isolated from the 2BS cells using an RNeasy Mini kit (QIAGEN, Gaithersburg, MD, USA) according to the manufacturer’s instructions and then subjected to reverse transcription using the StarScript first strand cDNA synthesis kit (Transgen Biotech, Beijing, China). Real-time PCR was performed in triplicate using the SYBR Green PCR Master Mix (Applied Biosystems, Foster City, CA, USA) on an ABI Prism 7500 sequence detector (Applied Biosystems). The *β-actin* gene served as an endogenous control for normalization. The primers were summarized in Supplementary Table 1.

### Western blotting

Whole cell extracts were generated by direct lysis with radioimmune precipitation assay (RIPA) buffer (Beyotime, Shanghai, China) with protease inhibitor mixture (Thermo Fisher Scientific). Protein concentration was determined by BCA Protein Assay Reagent (Thermo Fisher Scientific). Samples were boiled by addition 6 × SDS sample buffer for 10 min at 100 °C and 40 *μ*g of protein was utilized for each western blot. The primary antibodies used for western blot analysis were described as above. The intensity of the protein bands was quantified by densitometry using ImageJ software (NIH, Bethesda, MD, USA). Each western blot was repeated at least three times.

### ELISA

The supernatants of the 2BS cells were collected after treatment as indicated. IL-8 level was measured by an ELISA kit from Boster (Wuhan, China).

### ChIP assay

ChIP assays were performed using the Chromatin Immunoprecipitation Assay kit (Upstate, New York, NY, USA) according to manufacturer’s instruction. DNA released from the precipitated complexes was amplified by real-time PCR using sequence specific primers.

### SA-*β*-gal analysis

For SA-*β*-gal staining, the cells were washed twice with phosphate buffered saline (PBS), fixed for 3-5 min at room temperature in 3% formaldehyde and washed again with PBS. Cells were then incubated overnight at 37 °C without CO_2_ in a freshly prepared staining buffer.

### Cell proliferation assay

Growth curves were assayed using the 3-(4,5-dimethylthiazol-2-yl)-2,5-diphenyltetrazolium bromide (MTT) method. Cells were seeded into 96-well plates and cultured for periods. At the indicated time, an aliquot of cells were stained with 20 *μ*l of MTT reagent (5 mg/ml in PBS; M2128, Sigma) for 1 h and then treated with DMSO for 10 min. The optical density was measured at 570 nm.

### Colony formation

In order to determine the colony formation, cells were cultured in plates. Six days later, cells were fixed in 3% formaldehyde at 37 °C for 30 min and washed twice with 1 × PBS, then stained with crystal violet for 1 h and washed with 1 × PBS twice.

### Cell cycle analysis

When cells reached 70–80% confluence, they were washed with PBS, and then detached with 0.25% trypsin and fixed with 75% ethanol overnight. Thereafter, the cells were treated with 1 mg/ml RNase A (R6513, Sigma) at 37 °C for 30 min, resuspended in 0.5 ml of PBS and stained with propidium iodide in the dark for 30 min. Fluorescence was measured with a FACScan flow cytometry system (BD Biosciences, Lake Franklin, New Jersey, USA).

### Tumourigenic assay

EC109 and EC9706 cells were resuspended in 100 *μ*l PBS and 1 × 10^6^ cells were injected subcutaneously into the left or right side of each NOD/SCID mice (Beijing Vital River Laboratory Animal Technology Co., Ltd.). One side was implanted with control tumor cells, and the other with tested tumor cells. Tumor sizes were monitored every five days, and tumor volumes were calculated as volume=length × width^2^ × (1/2). Animals were maintained on regular food and water. All procedures were approved by the Institutional Animal Care and Use Committee.

### Statistical analysis

Data are presented as the means±S.D. Statistical difference between groups was analyzed by a Student *t*-test or one-way ANOVA tests where appropriate. Besides, a Bonferroni correction was done when multiple comparisons were performed. A *P*-value<0.05 was considered as statistically significant.
