# Supplementary material for: FOXQ1 regulates senescence-associated inflammation via activation of SIRT1 expression
Source: Cell Death Dis. 2017 Jul 20;8(7):e2946–. doi: 10.1038/cddis.2017.340 (PMC5550881; doi:10.1038/cddis.2017.340)
Supplement: Supplementary Table 1 [file cddis2017340x1.docx]

Table 1. Sequences of oligonucleotides used for qRT-PCR, ChIP-qPCR and RNA interference assay.

| Primers for qRT-PCR | Forward primer | Reverse primer |
| --- | --- | --- |
| IL-6 | TGACCCAACCACAAATGC | CTGGCTCTGAAACAAAGGAT |
| IL-8 | TGTGGGTCTGTTGTAGGG | GTGAGGTAAGATGGTGGC |
| SIRT1 | GATCTTCCAGATCCTCAAGCG | AGGACATCGAGGAACTACCTG |
| FOXQ1 | CGGAGATCAACGAGTACCTCA | GTTGAGCATCCAGTAGTTGTCCTT |
| β-actin | GTGGACATCCGCAAAGAC | AAAGGGTGTAACGCAACTAA |
| Primers for ChIP-qPCR | Forward primer | Reverse primer |
| SIRT1 | AGGAGCTGTCAGAACGGTGT | CGTGTAGTGCAGCCAAATTC |
| RNA interference assay | FOXQ1-siRNA | Scramble siRNA |
|  | AGATCAACGAGTACCTCAT | UUCUCCGAACGUGUCACGU |
